# Supplementary material for: High-quality listening behaviors linked to social connection between strangers
Source: Commun Psychol. 2025 Nov 21;3:165. doi: 10.1038/s44271-025-00342-2 (PMC12638239; doi:10.1038/s44271-025-00342-2)
Supplement: Supplementary file 2 — Supplemental material [file 44271_2025_342_MOESM2_ESM.pdf]

Supplemental Material for:

High-Quality Listening Behaviors Linked to Social Connection Between Strangers

Table of Contents

STUDY 1 3

I. Pre-registration 3

II. Scripts for Ellie, Virtual Human 4

III. Verbal listening Coding scheme 6

IV. Supplemental analyses 7

Table S1.1 Unadjusted models: Follow-up questions and conversational response times 7
Table S1.2 Unadjusted models: Verbal validation and conversational response times 7
Table S1.3 Unadjusted models: Positivity resonance and listening behaviors 8
Table S1.4 Unadjusted models: Social connectedness intervention effects on listening behaviors 8
Table S1.5. Listening behaviors and conversational response times (raw variables) 9
Table S1.6. Listening behaviors and positivity resonance (raw variables) 9
Table S1.7. Social connectedness intervention effects using raw variables 10
Figure S1.1 Confirmatory Factor Analysis of Social Connection 10

STUDY 2 11

V. Pre-registration 11

VI. Global Listening Coding Scheme 11

VII. Supplemental analyses 12

Table S2.1. Unadjusted models: Listening behaviors on conversational response times. 12
Table S2.2 Unadjusted models: Positivity resonance and listening behaviors 12
Table S2.3. Descriptives of listening behaviors by condition 13
Table S2. 4 Individual condition effects of social connectedness interventions on listening behaviors 13
Table S2.5. Listening behaviors and conversational response times (raw variables) 14
Table S2. 6 Positivity resonance and listening behaviors (raw variables) 15

Study 1

I. Pre-registration

https://osf.io/nh9j6/?view_only=9a4c08436c6d41e3b639c6d67c6c55ce

i. Deviations

a. Although we had pre-registered additionally assessing nonverbal indicators, attempts to code purely nonverbal cues of listening were aborted early in coding training (week 2 of 6) due to combined concerns about validity as well as reliability. Although reliability did not yet reach our threshold of ICC >.80 (nonverbal indicators average ICC of .75 and single-coder ICC of .37), our decision was ultimately driven by a loss of confidence in the nonverbal cues coded as valid measures of high-quality listening when considered in isolation. We therefore narrowed our focus to verbal indicators for Study 1, which have been argued to be the strongest behavioral signals of high-quality listening.

b. Under hypotheses, we preregistered "We will also explore the potential main effect of rapport features, and its interaction with the connection condition on listening behavior." However, given the original report (published after this study's preregistration; West et al., 2024) later reported minimal findings relating to nonverbal cue (i.e., rapport) condition, we no longer suspected it would influence listening behaviors. Even so, the preregistered exploratory main effect findings can be found in supplementary analyses reported below (Section IV), where this randomized variable is entered as a control. When testing as an interaction between intervention condition and nonverbal cue condition, no signification interactions emerge for follow up questions (β = -0.03, b = -0.08, 95% CI [-0.41, 0.24], p = .616) nor verbal validation (β = 0.02, b = 0.06, 95% CI [-0.26, 0.38], p = .701).

c. As noted in the manuscript, we had preregistered testing all hypotheses with multiple regression. Although appropriate for participants' data, this was the incorrect statistical approach for confederate/experimenter outcomes. Because confederates had repeated interactions (with different participants), to account for non-independence of observations, we tested effects of listening predictors on partner conversational response time and partner-reported positivity resonance using multi-level models.

II. Scripts for Ellie, Virtual Human

Ellie's scripts

Blue = phrase is the same across conditions

Connection

1. Hi there, I'm Ellie. What is your name?
2. Nice to meet you. Thanks for coming in today.
3. What did you think of that video?
4. Thanks for sharing.
5. The woman in the video thought moments of connection are really important.
6. Did you find the message compelling?
7. Have you experienced the kind of thing she was talking about? Really connecting with someone you barely knew?
    a. *If "No":* Maybe you could take a moment to imagine what that might be like.
8. Could you tell me more about what that experience is like? I don't get out much myself, you know.
9. The goal for this research study is to see what happens when people adopt positive behavior goals, like seeking out more positive connections with strangers and acquaintances.
10. Would you be willing to try to do that?
11. The video mentioned many benefits of engaging in this behavior, and there may be others as well. Can you imagine any benefits that you would value personally?
    a. *If "No":* I'm sorry to hear that you don't see the personal value in it. The researchers tell me there is a lot of evidence that positive connections with strangers and acquaintances can be beneficial, so I've been programmed to continue.
    b. *If "Yes" without elaboration*: Could you tell me more? What would you find personally beneficial about it?
12. I'd like to really encourage you to find more moments of connection with strangers and acquaintances over the next 24 hours.
13. You might try making more eye contact and smiling with people. You know, just be attentive to others and try to connect with them.
14. What does your day look like between now and this time tomorrow?

Breathing

1. Hi there, I'm Ellie. What is your name?
2. Nice to meet you. Thanks for coming in today.
3. What did you think of that video?
4. Thanks for sharing.
5. The woman in the video thought proper breathing is really important.
6. Did you find the message compelling?
7. Have you tried the thing she was talking about? Breathing from your belly, instead of your neck and shoulders?
    a. *If "No":* Maybe you could try it now.
8. Could you tell me more about what it feels like? I don't actually breathe myself, you know.
9. The goal for this research study is to see what happens when people adopt positive behavior goals, like breathing in a healthier way that is better aligned with human anatomy.
10. Would you be willing to try to do that?
11. The video mentioned many benefits of engaging in this behavior, and there may be others as well. Can you imagine any benefits that you would value personally?
    b. *If "No":* I'm sorry to hear that you don't see the personal value in it. The researchers tell me there is a lot of evidence that breathing expansively from your belly can be beneficial, so I've been programmed to continue.
    c. *If "Yes" without elaboration*: Could you tell me more? What would you find personally beneficial about it?
12. I'd like to really encourage you to find more moments to breathe from your belly over the next 24 hours.
13. You might try keeping your shoulders still and expanding your ribcage when you inhale. You know, just think about breathing expansively from your abdomen.
14. What does your day look like between now and this time tomorrow?
15. Could you find opportunities during that time to remind yourself to breathe properly?

15. Could you find opportunities during that time to connect with strangers and acquaintances?
16. Tell me more. Can you visualize and describe one of those opportunities?
17. Research has found that phrasing your plan as a statement that begins with the word IF, and includes the word THEN, can help you remember when and how to act on your intention.
18. Here's an example: IF I am sitting in class next to someone I don't know, THEN I will smile at them and introduce myself.
19. Could you try making your own? Use the opportunity you already described after the word IF, and identify the action you would take after the word THEN.
    a. *If response not in the right format:* Thanks for trying it out. To make this work well, be sure the phrase starting with IF describes a situation you might encounter, and the phrase starting with THEN describes your plan of action.
20. Now can you tell me about something that might keep you from seizing an opportunity to engage with a stranger or acquaintance?
21. Could you do anything to overcome that?
22. Can you try framing that in the same format as before, using the words IF and THEN?
23. Use the obstacle you described after the word IF, and say how you would overcome it after the word THEN.
24. Well, I've asked everything I need to. Thanks for sharing your thoughts with me!
25. Try to keep those opportunities and obstacles in mind.
26. I hope you have a great day, and enjoy connecting with people!
27. Goodbye!

16. Tell me more. Can you visualize and describe one of those opportunities?
17. Research has found that phrasing your plan as a statement that begins with the word IF, and includes the word THEN, can help you remember when and how to act on your intention.
18. Here's an example: IF I have a break between class, THEN I will focus on a few deep breaths that allow my back and belly to expand.
19. Could you try making your own? Use the opportunity you already described after the word IF, and identify the action you would take after the word THEN.
    a) *If response not in the right format:* Thanks for trying it out. To make this work well, be sure the phrase starting with IF describes a situation you might encounter, and the phrase starting with THEN describes your plan of action.
20. Now can you tell me about something that might keep you from seizing an opportunity to try breathing from your belly?
21. Could you do anything to overcome that?
22. Can you try framing that in the same format as before, using the words IF and THEN?
23. Use the obstacle you described after the word IF, and say how you would overcome it after the word THEN.
24. Well, I've asked everything I need to. Thanks for sharing your thoughts with me.
25. Try to keep those opportunities and obstacles in mind.
26. I hope you have a great day, and happy breathing!
27. Goodbye!

Other phrases

Yes, that's great.

Not quite.

Oops!

Hmm

I see.

One moment.

Oh. I am sorry to hear that.
Could you tell me any more about that?
Yes
No
OK
Sorry, I didn't catch that. Could you say that again?
Sure, I can say that again.
I am sorry, I don't know how to respond to that yet.
I am sorry, I am unable to tell you about that.
If you're having trouble, you can ask your experimenter for help.

III. Verbal listening Coding scheme

Instructions:
Verbal Indicators: For both verbal indicators, count the frequency the participant asks questions or expresses validation or understanding in each 30-second bin.

(1) Asks follow-up questions/exploring questions:
- Does not include question prompts or clarification questions about the prompt/ fast-friends task. Does not include general questions about the study or procedure.
- Listener asks questions that convey wanting to learn more or further understand either the topic that the speaker is discussing or about the speaker themselves
- Listeners' question is related to the topic the speaker is discussing and is not changing topics or not relevant to something the speaker said.
- Listener asks for clarification or repetition
- Listener asks about the speaker's feelings about the topic of conversation
- Listener states that they were going to ask a question ("I was going to ask about that")

(2) Expresses verbal validation or understanding:
- Does not include yeah/mhmms
- Does not include simply expressing agreement (e.g., I agree, same, me too)
- May include paraphrasing, reflecting information they heard from speaker, offering support, phrases that express understanding ("I get that", "gotcha", "really", "right", "sure", "I've heard of that")
- The listener exclaims an opinion or active interest about what the speaker is discussing—could be positive or negative ("wow", "jeez", "that's great")
- Count each unique statement, even if within the same response (e.g., "I can't believe that. You did the right thing" would be 2 counts).

IV. Supplemental analyses

Unadjusted models refer to models that are not adjusted for gender or confederate effects. Models are still adjusted for condition effects.

Hypothesis 1: Listening behaviors are associated with markers of social connection

Table S1.1 Unadjusted models: Follow-up questions and conversational response times

	Participant Response Time				Partner Response Time			
Predictors	*b*	*std. Beta*	*CI*	*p*	*b*	*std. Beta*	*CI*	*p*
(Intercept)	0.74	0.00	0.67, 0.82	**<0.001**	0.64	-0.07	0.58, 0.71	**<0.001**
Follow-up Questions	-0.11	-0.25	-0.16, -0.06	**<0.001**	-0.08	-0.24	-0.36, -0.12	**<0.001**
Nonverbal cue condition	-0.05	-0.08	-0.13, 0.02	0.164	-0.05	-0.09	-0.10, 0.01	0.120
Intervention condition	-0.01	-0.01	-0.08, 0.07	0.838	-0.00	-0.00	-0.12, 0.11	0.952

Note. Binary condition variables were coded as: nonverbal cue condition, on = 1 and off = 0; intervention condition, connection = 1 and active control = 0. Unstandardized 95% CI displayed.

Table S1.2 Unadjusted models: Verbal validation and conversational response times

	Participant Response Time				Partner Response Time			
Predictors	*b*	*std. Beta*	*CI*	*p*	*b*	*std. Beta*	*CI*	*p*
(Intercept)	1.05	0.00	0.94, 1.16	**<0.001**	0.78	-0.06	0.69, 0.88	**<0.001**
Verbal validation	-0.21	-0.45	-0.26, -0.16	**<0.001**	-0.11	-0.29	-0.15, -0.07	**<0.001**
Nonverbal cue condition	-0.05	-0.07	-0.11, 0.02	0.193	-0.04	-0.09	-0.10, 0.01	0.131

Intervention condition	-0.01	-0.02	-0.08, 0.06	0.766	-0.01	-0.01	-0.06, 0.05	0.834

Note. Binary condition variables were coded as: nonverbal cues condition, on = 1 and off = 0; intervention condition, connection = 1 and active control = 0. Unstandardized 95% CI displayed.

Table S1.3 Unadjusted models: Positivity resonance and listening behaviors

Model	Listening behavior	*B*	b	95% CI
Participant Positivity Resonance	Follow-up questions	0.03	0.07	-0.18, 0.31
	Verbal validation	0.07	0.15	-0.11, 0.40
Partner Positivity Resonance	Follow-up questions	0.22***	0.49	0.22, 0.76
	Verbal validation	0.34***	0.81	0.54, 1.08
BIPR	Follow-up questions	0.17*	0.16	0.05, 0.28
	Verbal validation	0.24***	0.25	0.13, 0.37

Note. BIPR = behavioral indicators of positivity resonance. Standardized betas with associated significance levels (****p* < .001, ** *p* < .01, **p* < .05). Unstandardized betas and associated 95% confidence intervals are also presented. Adjusted for condition effects.

Hypothesis 2: Social connectedness interventions increase listening behaviors

Table S1.4 Unadjusted models: Social connectedness intervention effects on listening behaviors

	**Follow-up questions**				**Verbal validation**			
Predictors	*b*	*std. Beta*	*CI*	*p*	*b*	*std. Beta*	*CI*	*p*
(Intercept)	0.66	0.00	0.52, 0.81	**<0.001**	1.83	-0.00	1.69, 1.97	**<0.001**

Nonverbal cue condition	-0.07	-0.05	-0.24, 0.10	0.398	-0.02	-0.01	-0.18, 0.14	0.820
Intervention condition	0.23	0.16	0.06, 0.40	**0.007**	0.10	0.07	-0.06, 0.26	0.226

Note. Binary condition variables were coded as: nonverbal cue condition, on = 1 and off = 0; intervention condition, connection = 1 and active control = 0. Unstandardized 95% CI displayed.

Study 1 model results using raw variables (non-winsorized and non-transformed).

Hypothesis 1: Listening behaviors are associated with markers of social connection

Table S1.5. Listening behaviors and conversational response times (raw variables)

	**Participant Response Time**	**Partner Response Time**
**Follow-up Questions**	β = -0.18, *b* = -0.05<br>95% CI [-0.09, -0.02], *p* = .006	β = -0.16, *b* = -0.04<br>95% CI [-0.07, -0.01], *p* = .008
**Verbal Validation**	β = -0.34, *b* = -0.04<br>95% CI [-0.06, -0.03], *p* < .001	β = -0.24, *b* = -0.03<br>95% CI [-0.04, -0.01], *p* < .001

Note. All models controlled for condition, gender, and experimenter.

Table S1.6. Listening behaviors and positivity resonance (raw variables)

Model	Listening behavior	*B*	b	95% CI
Participant Positivity Resonance	Follow-up questions	0.03	0.01	-0.05, 0.08
	Verbal validation	-.04	-0.01	-0.04, 0.02
Partner Positivity Resonance	Follow-up questions	0.12*	0.07	0.01, 0.14
	Verbal validation	0.22***	0.06	0.03, 0.09
	Follow-up questions	0.12*	0.14	0.01, 0.27

BIPR	Verbal validation	0.28***	0.15	0.09, 0.21

Note. BIPR = behavioral indicators of positivity resonance. Standardized betas with associated significance levels (***$p < .001$, ** $p < .01$, *$p < .05$). Unstandardized betas and associated 95% confidence intervals are also presented. Adjusted for condition effects, gender and experimenter.

Table S1.7. Social connectedness intervention effects using raw variables

	**Follow-up questions**	**Verbal validation**
**Intervention condition**	$\beta = 0.14$, $b = 0.80$<br>95% CI [0.03, 0.25], $p = .013$	$\beta = 0.05$, $b = 0.54$<br>95% CI [-0.80, 1.88], $p = .425$

Note. Models controlled for nonverbal cues condition, gender and experimenter.

Figure S1.1 Confirmatory Factor Analysis of Social Connection

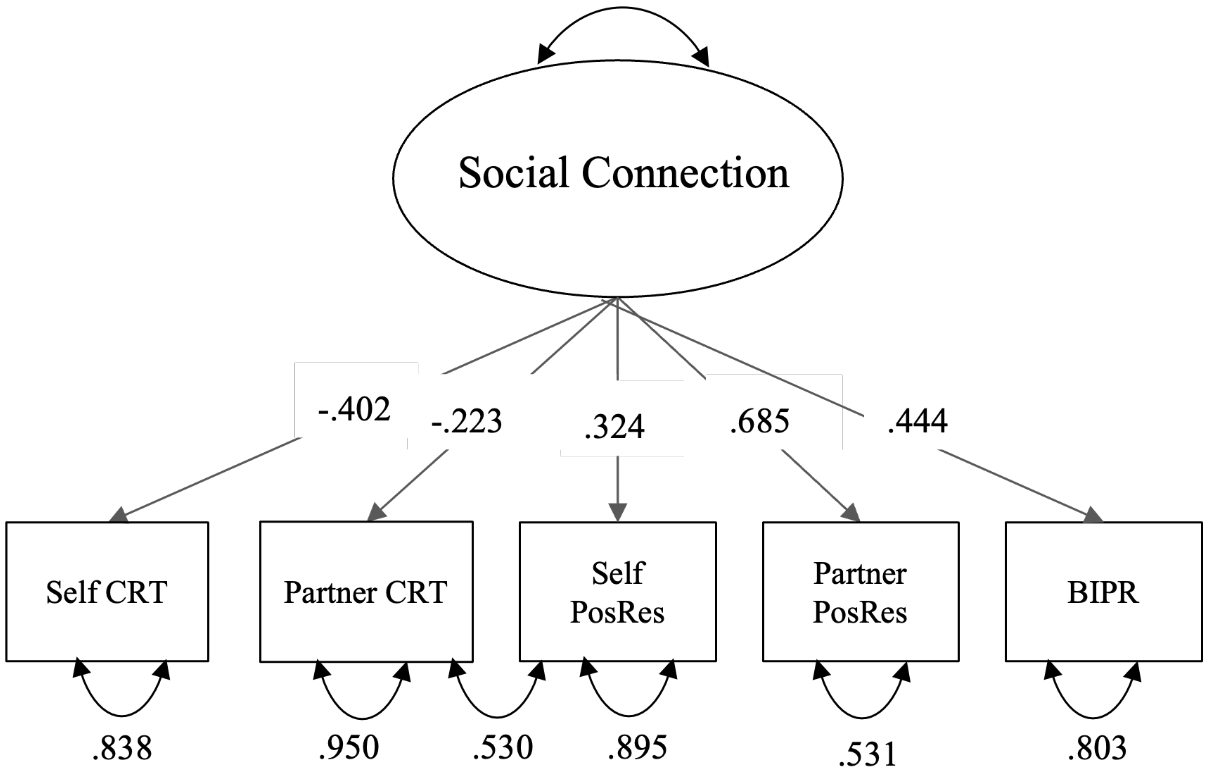

Note. Confirmatory factor analysis of prosocial tendencies. Standardized coefficient estimates are reported. All factor loadings were significant (*p*s < .01). CRT = conversational response time, PosRes = positivity resonance, BIPR = behavioral indicators of positivity resonance.

STUDY 2

V. Pre-registration

https://osf.io/xz39m/?view_only=c07943eee8a14dddb159e1b43c085405

i. Deviations

a. As noted in the manuscript, we had originally preregistered under hypotheses "Participants in either of the social connection conditions will engage in more listening behaviors compared to the passive control group." However, given results from the original research report on this data showed that mindfulness (the active control) increased perceived positivity resonance to a comparable degree as the two social connectedness interventions (Zhou et al., 2022), consistent with other published secondary analyses of these same data (Prinzing et al., 2023), we grouped the three active conditions together to compare against the passive control group. Results of the individual condition effects for the social connectedness intervention can be found in Supplemental Table S2.3.

b. As noted in the manuscript, and as in Study 1, we had preregistered testing all hypotheses with multiple regression. Although appropriate for participants' data, this was the incorrect statistical approach for confederate/experimenter outcomes. Because experimenters had repeated interactions (with different participants), to account for non-independence of observations, we tested effects of listening predictors on partner conversational response time and partner-reported positivity resonance using multi-level models.

VI. Global Listening Coding Scheme

Instructions:

High-quality Listening Definition:

Listening is a multi-faceted but ultimately holistic process that includes attention, comprehension, and positive regard (Kluger & Itzchakov, 2022).

- Attention is conveyed through effective backchannel behaviors (verbal or non-verbal reactions), such as nodding, maintaining eye contact, orienting the speaker's body posture and vocal utterances such as mhmm and yea.
- Comprehension is conveyed by paraphrasing the speaker's message and asking open questions relevant to the speaker's stories.
- Positive regard is conveyed by exhibiting conversational receptiveness, including the use of hedges; that is, phrases such as "somewhat" and "might," which soften the tone of the conversation and indicate a non-judgmental attitude.

While viewing the video clip, please answer the following question for each 30- second time interval:

"Did the participant exhibit high-quality listening? That is, did they show attention via backchannel behaviors, comprehension through paraphrasing and asking open questions, or convey positive intention?"

Base your coding on the overall feeling you get while watching the video segments.
Use the following 1 to 5-point scale based on intensity, duration and clarity of behaviors:
0= None or very brief
1= A little bit (a few times or at low intensity)
2= A moderate amount (e.g., one or more behaviors displayed, maybe half the time or sometimes with low intensity – felt "average")
3= More than a moderate amount but not quite a 4
4 = A lot (e.g. multiple behaviors displayed for a majority of the time and at high intensity)

VII. Supplemental analyses

Unadjusted models refer to models that are not adjusted for gender or experimenter effects. Models are still adjusted for condition effects. The length of the video (quantified by the number of coding bins) was additionally controlled for in analyses testing global listening behaviors.

Hypothesis 1: Listening behaviors are associated with markers of social connection

Table S2.1. Unadjusted models: Listening behaviors on conversational response times.

	**Participant Response Time**	**Partner Response Time**
**Global**	β = -0.16, *b* = -0.05<br>95% CI [-0.08, -0.02], *p* = .005	β = -0.16, *b* = -0.05<br>95% CI [-0.09, -0.02], *p* = .003
**Verbal Validation (log)**	β = -0.21, *b* = -0.11<br>95% CI [-0.17, -0.05], *p* < .001	β = -0.10, *b* = -0.06<br>95% CI [-0.12, 0.01], *p* = .079
**Follow-up Questions (log)**	β = 0.01, *b* = 0.01<br>95% CI [-0.10, 0.12], *p* = .833	β = -0.18, *b* = -0.18<br>95% CI [-0.29, -0.07], *p* = .001

Note. The length of the video (quantified by the number of coding bins) was additionally controlled for in analyses testing global listening behaviors. Each cell represents an independent model, controlling for condition effects.

Table S2.2 Unadjusted models: Positivity resonance and listening behaviors

Model	Listening behavior	*B*	b	95% CI
Partner Positivity Resonance	Follow-up questions	0.05	0.49	-0.53, 1.51
	Verbal validation	0.26***	1.41	0.84, 1.98
	Global	0.21***	0.65	0.35, 0.95
BIPR	Follow-up questions	-0.02	-0.03	-0.23, 0.16
	Verbal validation	0.19***	0.19	0.09, 0.30
	Global	0.32***	0.19	0.13, 0.25

Note. BIPR = behavioral indicators of positivity resonance. Standardized betas with associated significance levels (****p* < .001, ** *p* < .01, **p* < .05). Unstandardized betas and associated 95% confidence intervals are also presented. Adjusted for condition effects.

Hypothesis 2: Social connectedness interventions increase listening behaviors

Table S2.3. Descriptives of listening behaviors by condition

**30-sec Bin Average**	**Behavioral Intervention Group**	**Passive Control Group**
	M (SD)	***M (SD)***
Global	1.48 (0.56)	1.47 (0.53)
Follow-up Questions	0.33 (0.26)	0.29 (0.24)
Verbal Validation	1.13 (0.75)	1.04 (0.64)

Table S2. 4 Individual condition effects of social connectedness interventions on listening behaviors

	**Follow-up questions**			**Verbal validation**			**Global**		
Predictors	*std. Beta*	*CI*	*p*	*std. Beta*	*CI*	*p*	*std. Beta*	*CI*	*p*

(Intercept)	-0.09	-0.30,0.12	**<0.001**	0.04	-0.16, 0.25	**<0.001**	0.10	-0.11, 0.30	**0.002**
Social connectedness-Weak tie	0.14	-0.16, 0.44	0.359	0.13	-0.16, 0.41	0.391	-0.04	-0.33, 0.25	0.807
Mindfulness	0.09	-0.21, 0.39	0.545	0.12	-0.17, 0.41	0.426	-0.01	-0.30, 0.28	0.949
Social connectedness - general	0.30	0.00, 0.60	**0.049**	0.09	-0.20, 0.38	0.536	0.07	-0.23, 0.36	0.652
Gender [women]	-0.04	-0.15, 0.06	0.428	-0.00	-0.10, 0.10	0.978	0.03	-0.07, 0.13	0.572
Experimenter [2]	-0.04	-0.50, 0.42	0.866	-0.62	-1.06, -0.17	**0.006**	-0.51	-0.98, -0.05	**0.030**
Experimenter [3]	-0.27	-0.77, 0.22	0.276	-1.10	-1.58, -0.63	**<0.001**	-1.26	-1.77, -0.76	**<0.001**
Experimenter [4]	-0.35	-0.75, 0.05	0.090	-0.49	-0.88, -0.09	**0.015**	-0.14	-0.53, 0.25	0.485
Length/ Bin count							0.12	0.01, 0.23	**0.036**

Note. Passive control serves as the reference group. Standardized CI's presented.

Study 2 model results using raw variables (non-winsorized and non-transformed).

Hypothesis 1: Listening behaviors are associated with markers of social connection

Table S2.5. Listening behaviors and conversational response times (raw variables)

	**Participant Response Time**	**Partner Response Time**
**Global**	β = -0.10, *b* = -0.06<br>95% CI [-0.12, 0.01], *p* = .078	β = -0.16, *b* = -0.09<br>95% CI [-0.15, -0.03], *p* = .004
**Verbal Validation**	β = -0.16, *b* = -0.07<br>95% CI [-0.12, -0.02], *p* = .007	β = -0.13, *b* = -0.05<br>95% CI [-0.10, -0.01], *p* = .024
**Follow up Questions**	β = -0.02, *b* = -0.02	β = -0.15, *b* = -0.17

	95% CI [-0.15, 0.11], *p* = .776	95% CI [-0.30, -0.05], *p* = .007

Table S2. 6 Positivity resonance and listening behaviors (raw variables)

Model	Listening behavior	*B*	b	95% CI
Partner Positivity Resonance	Follow-up questions	0.05	0.31	-0.39, 1.02
	Verbal validation	0.26***	0.61	0.37, 0.86
BIPR	Follow-up questions	-0.02	-0.03	-0.17, 0.10
	Verbal validation	0.18***	0.08	0.03, 0.13

Note. Adjusted for gender and experimenter effects. Global variable is not included here because it is reported in the primary manuscript using non-winsorized, non-transformed scores.

References

Kluger, A. N., & Itzchakov, G. (2022). The power of listening at work. *Annual Review of Organizational Psychology and Organizational Behavior, 9*(1), 121-146.

Prinzing, M., Le Nguyen, K., & Fredrickson, B. L. (2023). Does shared positivity make life more meaningful? Perceived positivity resonance is uniquely associated with perceived meaning in life. *Journal of Personality and Social Psychology, 125*(2), 345–366. https://doi.org/10.1037/pspi0000418

West, T. N., Prinzing, M. M., Garton, C., Berman, C. J., Zhou, J., Hale, J., ... & Fredrickson, B. L. (2024). Improving social connection with weak ties and strangers: effects of a new micro-intervention on interaction quality and social behavior. *The Journal of Positive Psychology*, 1-11.

Zhou, J., Prinzing, M. M., Le Nguyen, K. D., West, T. N., & Fredrickson, B. L. (2022). The goods in everyday love: Positivity resonance builds prosociality. *Emotion*, *22*(1), 30–45. https://doi.org/10.1037/emo0001035
