# Supplementary material for: High-quality listening behaviors linked to social connection between strangers
Source: Commun Psychol. 2025 Nov 21;3:165. doi: 10.1038/s44271-025-00342-2 (PMC12638239; doi:10.1038/s44271-025-00342-2)
Supplement: Supplementary file 3 — Reporting summary [file 44271_2025_342_MOESM3_ESM.pdf]

nature portfolio

Corresponding author(s): Taylor West

Last updated by author(s): Jul 15, 2025

Reporting Summary

Nature Portfolio wishes to improve the reproducibility of the work that we publish. This form provides structure for consistency and transparency in reporting. For further information on Nature Portfolio policies, see our Editorial Policies and the Editorial Policy Checklist.

Statistics

For all statistical analyses, confirm that the following items are present in the figure legend, table legend, main text, or Methods section.

n/a	Confirmed	
☐	☒	The exact sample size (*n*) for each experimental group/condition, given as a discrete number and unit of measurement
☐	☒	A statement on whether measurements were taken from distinct samples or whether the same sample was measured repeatedly
☐	☒	The statistical test(s) used AND whether they are one- or two-sided<br>*Only common tests should be described solely by name; describe more complex techniques in the Methods section.*
☐	☒	A description of all covariates tested
☐	☒	A description of any assumptions or corrections, such as tests of normality and adjustment for multiple comparisons
☐	☒	A full description of the statistical parameters including central tendency (e.g. means) or other basic estimates (e.g. regression coefficient) AND variation (e.g. standard deviation) or associated estimates of uncertainty (e.g. confidence intervals)
☐	☒	For null hypothesis testing, the test statistic (e.g. *F*, *t*, *r*) with confidence intervals, effect sizes, degrees of freedom and *P* value noted<br>*Give P values as exact values whenever suitable.*
☒	☐	For Bayesian analysis, information on the choice of priors and Markov chain Monte Carlo settings
☐	☒	For hierarchical and complex designs, identification of the appropriate level for tests and full reporting of outcomes
☐	☒	Estimates of effect sizes (e.g. Cohen's *d*, Pearson's *r*), indicating how they were calculated

Our web collection on statistics for biologists contains articles on many of the points above.

Software and code

Policy information about availability of computer code

Data collection: Data were collected using Qualtrics. Audio transcription was completed using AssemblyAI.

Data analysis: Data were analyzed using R version 4.3.1.

For manuscripts utilizing custom algorithms or software that are central to the research but not yet described in published literature, software must be made available to editors and reviewers. We strongly encourage code deposition in a community repository (e.g. GitHub). See the Nature Portfolio guidelines for submitting code & software for further information.

Data

Policy information about availability of data

All manuscripts must include a data availability statement. This statement should provide the following information, where applicable:

- Accession codes, unique identifiers, or web links for publicly available datasets
- A description of any restrictions on data availability
- For clinical datasets or third party data, please ensure that the statement adheres to our policy

We report how we determined our samples sizes, all data exclusions and manipulations used in both studies. All data and code are publicly available at [https://osf.io/2xbwt/?view_only=25e76a885eee4b8db51342b893ac0f78].

Research involving human participants, their data, or biological material

Policy information about studies with human participants or human data. See also policy information about sex, gender (identity/presentation), and sexual orientation and race, ethnicity and racism.

Reporting on sex and gender	Participants self-reported their gender. Gender was used as a covariate in sensitivity analyses to account for potential variance due to gender identity, particularly in cross-gender interactions, but we did not conduct gender-based analyses as gender was not of particular interest to the current study.
Reporting on race, ethnicity, or other socially relevant groupings	Participants self-reported on their race and ethnicity. This information was only used for describing sample characteristics.
Population characteristics	See above.
Recruitment	Participants in both sample were originally recruited from University of North Carolina at Chapel Hill in exchange for course credit (Study 1 only) or cash compensation (Study 1 and 2). Study 1 participants were between the ages of 18-35, and consisted of undergraduate and graduate students. Study 2 was a sample recruited from campus and the surrounding community. Participants must have been between the ages of 20 and 65 and not currently enrolled as an undergraduate student.
Ethics oversight	University of North Carolina at Chapel Hill

Note that full information on the approval of the study protocol must also be provided in the manuscript.

Field-specific reporting

Please select the one below that is the best fit for your research. If you are not sure, read the appropriate sections before making your selection.

☐ Life sciences ☒ Behavioural & social sciences ☐ Ecological, evolutionary & environmental sciences

For a reference copy of the document with all sections, see nature.com/documents/nr-reporting-summary-flat.pdf

Behavioural & social sciences study design

All studies must disclose on these points even when the disclosure is negative.

Study description	The currents studies are secondary analyses of two previously collected and reported interventions. Both studies are quantitative in nature and use experimental designs.
Research sample	Study 1 analyzed sample: Undergraduate and graduate students (N = 300), 75% Women, Mean Age = 20.02, SD = 2.63. Participants identified as either White (50%), Black (4.7%), Asian (25.5%) or Hispanic (10.4%), with the remaining 9.4% identifying as either multiracial, other, or preferred not to say.<br>Study 2 analyzed sample: Community sample N = 348; Mean age = 34.02, SD = 11.28. The sample was primarily female (81%), with a majority racially identifying as White (69.8%), followed by Black or African American (11.6%), Asian (8.2%), Hispanic or Latin American (7.3%), or other (3.1%).
Sampling strategy	Samples were recruited via convenience and undergraduate research pools, with sample size determined by the original aims associated with each study. We reported sensitivity power analyses in the manuscript.
Data collection	Data were collected using qualtrics and in-lab audio/video equipment. Participants interacted with a confederate (Study 1) or experimenter (Study 2), both blinded to participant's condition.
Timing	In Study 1, data collection occurred between Fall 2021 to Winter 2022. In Study 2, data collection occurred between March and November 2019.
Data exclusions	All data exclusions are reported in the manuscript.
Non-participation	We report any data exclusions relevant to our secondary analyses in the manuscript. The number of people who chose not to participant in the original studies are reported elsewhere.
Randomization	Participants were randomized to conditions in both studies.

Reporting for specific materials, systems and methods

We require information from authors about some types of materials, experimental systems and methods used in many studies. Here, indicate whether each material, system or method listed is relevant to your study. If you are not sure if a list item applies to your research, read the appropriate section before selecting a response.

Materials & experimental systems

n/a	Involved in the study
☒	☐ Antibodies
☒	☐ Eukaryotic cell lines
☒	☐ Palaeontology and archaeology
☒	☐ Animals and other organisms
☒	☐ Clinical data
☒	☐ Dual use research of concern
☒	☐ Plants

Methods

n/a	Involved in the study
☒	☐ ChIP-seq
☒	☐ Flow cytometry
☒	☐ MRI-based neuroimaging

Plants

Seed stocks

Report on the source of all seed stocks or other plant material used. If applicable, state the seed stock centre and catalogue number. If plant specimens were collected from the field, describe the collection location, date and sampling procedures.

Novel plant genotypes

Describe the methods by which all novel plant genotypes were produced. This includes those generated by transgenic approaches, gene editing, chemical/radiation-based mutagenesis and hybridization. For transgenic lines, describe the transformation method, the number of independent lines analyzed and the generation upon which experiments were performed. For gene-edited lines, describe the editor used, the endogenous sequence targeted for editing, the targeting guide RNA sequence (if applicable) and how the editor was applied.

Authentication

Describe any authentication procedures for each seed stock used or novel genotype generated. Describe any experiments used to assess the effect of a mutation and, where applicable, how potential secondary effects (e.g. second site T-DNA insertions, mosiacism, off-target gene editing) were examined.
